# 1-Ferrocenylmeth­yl-1*H*-imidazole

**DOI:** 10.1107/S1600536808029231

**Published:** 2008-10-22

**Authors:** Vincent O. Nyamori, Muhammad D. Bala

**Affiliations:** a School of Chemistry, University of KwaZulu-Natal, Westville Campus, Private Bag X54001, Durban 4000, South Africa

## Abstract

In the title compound, [Fe(C_5_H_5_)(C_9_H_9_N_2_)], the distances of the Fe atom from the centroids of the unsubstituted and the substituted cyclo­penta­dienyl (cp) rings are 1.639 (1) and 1.647 (1) Å, respectively. The ferrocenyl unit deviates from an eclipsed geometry with tilted cp rings; the inter­planar angle between the cp and imidazole rings is 114.11 (4)°.

## Related literature

For a related structure, see: Hua *et al.* (2004 ▶). For applications of aryl­imidazoles, see: Broggini & Togni (2002 ▶); César *et al.* (2004 ▶); Cozzi *et al.* (1993 ▶); Herrmann & Köcher (1997 ▶); Lee & Nolan (2000 ▶); Ohmori *et al.* (1996 ▶); Snegur *et al.* (2004 ▶).
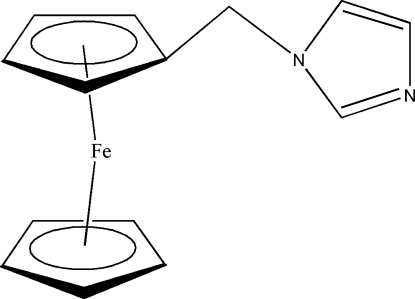


## Experimental

### 

#### Crystal data

[Fe(C_5_H_5_)(C_9_H_9_N_2_)]
*M* *_r_* = 266.12
Monoclinic, 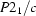

*a* = 14.8914 (6) Å
*b* = 7.5587 (3) Å
*c* = 10.7854 (4) Å
β = 96.862 (2)°
*V* = 1205.30 (8) Å^3^
*Z* = 4
Mo *K*α radiation
μ = 1.23 mm^−1^
*T* = 293 (2) K
0.39 × 0.26 × 0.05 mm

#### Data collection

Bruker APEXII CCD area-detector diffractometer
Absorption correction: integration (*XPREP*; Bruker, 2005 ▶) *T*_min_ = 0.646, *T*_max_ = 0.941
17808 measured reflections
2904 independent reflections
1852 reflections with *I* > 2σ(*I*)
*R*_int_ = 0.054

#### Refinement

*R*[*F* ^2^ > 2σ(*F* ^2^)] = 0.040
*wR*(*F* ^2^) = 0.114
*S* = 1.00
2904 reflections
154 parameters
H-atom parameters constrained
Δρ_max_ = 0.63 e Å^−3^
Δρ_min_ = −0.38 e Å^−3^

### 

Data collection: *APEX2* (Bruker, 2005 ▶); cell refinement: *SAINT-Plus* (Bruker, 2005 ▶); data reduction: *SAINT-Plus*; program(s) used to solve structure: *SHELXTL* (Sheldrick, 2008 ▶); program(s) used to refine structure: *SHELXTL*; molecular graphics: *PLATON* (Spek, 2003 ▶) and *ORTEP-3* (Farrugia, 1997 ▶); software used to prepare material for publication: *SHELXTL*.

## Supplementary Material

Crystal structure: contains datablocks global, I. DOI: 10.1107/S1600536808029231/dn2373sup1.cif

Structure factors: contains datablocks I. DOI: 10.1107/S1600536808029231/dn2373Isup2.hkl

Additional supplementary materials:  crystallographic information; 3D view; checkCIF report

## Figures and Tables

**Fig. 1.**
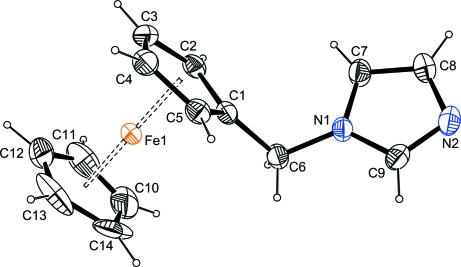
Molecular structure of the title complex with the atom labelling scheme. Ellipsoids are drawn at the 50% probability level.

## supplementary crystallographic information

### Comment

The synthesis of arylimidazole compounds is of importance because of their significance in pharmaceutical (Ohmori *et al.* 1996), biological (Cozzi *et al.* 1993) and the synthesis of fine chemicals (Lee & Nolan, 2000; Herrmann & Köcher, 1997). Ferrocenyl compounds with an *N*-heterocycle group such as ferrocenylmethyl benzimidazole have been studied and found to exhibit anticancer activity (Snegur *et al.* 2004). The ferrocenylimidazolium salts have also found uses in catalysis as precursors for the synthesis of *N*-heterocyclic carbenes (César *et al.*, 2004; Broggini & Togni, 2002).

In the title compound (I, Fig. 1), the distance of the Fe atom from the centroids of the substituted (C1—C5) and the unsubstituted (C6—C10) cyclopentadienyl rings are 1.639 (1) and 1.647 (1) Å respectively, indicating a slight shotening of the substituted cp—Fe bond length due to the substitution of the imidazole unit. The plane of the imidazole ring in (I) is tilted at an angle of 114.11 (4)° away from the plane of the C1—C5 cp ring. The strain on the substituted ring results in a corresponding tilt of 3.87 (2)° between the planes of the two cp rings. The cp rings also deviate significantly from an eclipsed conformation with torsion angles ranging from 19.98 (2)–24.90 (2)°. This could be due to the fact that the C1—C5 cp ring twists in order to accommodate the bulky imidazole unit, hence putting it out of coplanarity with the unsubstituted C10—C14 cp ring.

### Experimental

A mixture of equimolar amounts of ferrocenylmethanol (501 mg, 2.34 mmol) and *N,N'*-carbonyldiimidazole (379 mg, 2.34 mmol) in anhydrous dichloromethane was heated under reflux for 1 h. The resulting mass was cooled and then diethyl ether (50 cm^3^) was added and the resultant solution was allowed to stir for 3 minutes before being transferred to a separating funnel. The reaction mixture was then flushed with phosphoric acid (2 *x* 50 cm^3^). The aqueous phase fractions were then combined and the pH of the solution was adjusted to 5 using dilute sodium hydroxide. The aqueous solution was then extracted using dichloromethane (3 *x* 50 cm^3^). The dichloromethane extracts were combined, dried over anhydrous sodium sulfate, filtered and the solvent was removed in *vacuo*. The resulting product was subjected to column chromatography on a column of silica gel. Diethyl either was used to elute unreacted starting material and a mixture of ethyl acetate and methanol provided the title compound 1-(Ferrocenymethyl)-1*H*-imidazole. *Yield*: (398 mg, 64%); Yellow crystals mp 66–67 °C; IR *ν*_max_(KBr cm^-1^)3095, 1644, 1511, 1463, 1439, 1391, 1336, 1322,1279, 1238, 1221, 1104, 1079, 1040, 1027, 1002, 916, 811, 744, 752, 697, 662, 503, 482; ^1^H-NMR (CDCl_3_, 300 MHz) 7.50 (1*H*, s, NCH), 7.06 (1*H*, s, NCH), 6.94 (1*H*, s, NCH),4.88 (2*H*, s, CH_2_), 4.20 (4*H*, s, C_5_H_4_); 4.17(5*H*, s,*C*_5_H_5_); ^13^C-NMR (CDCl_3_, 300 MHz), 137.25, 129.67, 119.36, 83.09, 69.19, 69.16,68.93, 47.15; EI–MS 70 eV *m/z* 266 (*M*^+^,100%), 200 (12), 199 (70), 188 (23), 120 (52); (Found: [*M*^+^],266.050638. C_14_H_14_N_2_Fe requires [*M*^+^],266.050668).

### Refinement

All H atoms attached to C atoms were fixed geometrically and treated as riding with C—H = 0.95 Å and *U*_iso_(H) = 1.2*U*_eq_(C).

### Crystal data

**Table:**

[Fe(C_5_H_5_)(C_9_H_9_N_2_)]	*F*(000) = 552
*M**_r_* = 266.12	*D*_x_ = 1.467 Mg m^−^^3^
Monoclinic, *P*2_1_/*c*	Mo *K*α radiation, λ = 0.71073 Å
Hall symbol: -P 2ybc	Cell parameters from 3993 reflections
*a* = 14.8914 (6) Å	θ = 2.8–26.6°
*b* = 7.5587 (3) Å	µ = 1.23 mm^−^^1^
*c* = 10.7854 (4) Å	*T* = 293 K
β = 96.862 (2)°	Plate, yellow
*V* = 1205.30 (8) Å^3^	0.39 × 0.26 × 0.05 mm
*Z* = 4	

### Data collection

**Table:**

Bruker APEXII CCD area-detector diffractometer	2904 independent reflections
Radiation source: fine-focus sealed tube	1852 reflections with *I* > 2σ(*I*)
graphite	*R*_int_ = 0.054
φ and ω scans	θ_max_ = 28.0°, θ_min_ = 1.4°
Absorption correction: integration (XPREP; Bruker, 2005)	*h* = −19→18
*T*_min_ = 0.646, *T*_max_ = 0.941	*k* = −9→9
17808 measured reflections	*l* = −14→14

### Refinement

**Table:**

Refinement on *F*^2^	Primary atom site location: structure-invariant direct methods
Least-squares matrix: full	Secondary atom site location: difference Fourier map
*R*[*F*^2^ > 2σ(*F*^2^)] = 0.040	Hydrogen site location: inferred from neighbouring sites
*wR*(*F*^2^) = 0.114	H-atom parameters constrained
*S* = 1.00	*w* = 1/[σ^2^(*F*_o_^2^) + (0.0573*P*)^2^ + 0.4185*P*] where *P* = (*F*_o_^2^ + 2*F*_c_^2^)/3
2904 reflections	(Δ/σ)_max_ = 0.015
154 parameters	Δρ_max_ = 0.63 e Å^−^^3^
0 restraints	Δρ_min_ = −0.38 e Å^−^^3^

### Special details

**Table:**

Geometry. All e.s.d.'s (except the e.s.d. in the dihedral angle between two l.s. planes) are estimated using the full covariance matrix. The cell e.s.d.'s are taken into account individually in the estimation of e.s.d.'s in distances, angles and torsion angles; correlations between e.s.d.'s in cell parameters are only used when they are defined by crystal symmetry. An approximate (isotropic) treatment of cell e.s.d.'s is used for estimating e.s.d.'s involving l.s. planes.
Refinement. Refinement of *F*^2^ against ALL reflections. The weighted *R*-factor *wR* and goodness of fit *S* are based on *F*^2^, conventional *R*-factors *R* are based on *F*, with *F* set to zero for negative *F*^2^. The threshold expression of *F*^2^ > σ(*F*^2^) is used only for calculating *R*-factors(gt) *etc*. and is not relevant to the choice of reflections for refinement. *R*-factors based on *F*^2^ are statistically about twice as large as those based on *F*, and *R*- factors based on ALL data will be even larger.

### Fractional atomic coordinates and isotropic or equivalent isotropic displacement parameters (Å^2^)

**Table:**

	*x*	*y*	*z*	*U*_iso_*/*U*_eq_	
C1	0.74936 (18)	−0.0973 (4)	0.4338 (2)	0.0367 (6)	
C2	0.7628 (2)	−0.2266 (4)	0.3419 (3)	0.0426 (7)	
H2	0.7177	−0.2780	0.2863	0.051*	
C3	0.8561 (2)	−0.2636 (4)	0.3494 (3)	0.0464 (7)	
H3	0.8832	−0.3434	0.2996	0.056*	
C4	0.9012 (2)	−0.1589 (4)	0.4453 (3)	0.0465 (7)	
H4	0.9633	−0.1568	0.4695	0.056*	
C5	0.8361 (2)	−0.0576 (4)	0.4985 (3)	0.0412 (7)	
H5	0.8477	0.0216	0.5645	0.049*	
C6	0.6610 (2)	−0.0148 (4)	0.4525 (3)	0.0473 (7)	
H6A	0.6717	0.1044	0.4841	0.057*	
H6B	0.6239	−0.0065	0.3724	0.057*	
C7	0.5876 (2)	−0.2876 (4)	0.5291 (3)	0.0520 (8)	
H7	0.6011	−0.3673	0.4683	0.062*	
C8	0.5399 (2)	−0.3206 (5)	0.6267 (3)	0.0517 (8)	
H8	0.5150	−0.4296	0.6434	0.062*	
C9	0.5771 (2)	−0.0511 (4)	0.6399 (3)	0.0483 (8)	
H9	0.5835	0.0657	0.6667	0.058*	
C10	0.7592 (4)	0.1645 (8)	0.1970 (6)	0.104 (2)	
H10	0.6964	0.1723	0.1886	0.125*	
C11	0.8049 (4)	0.0625 (7)	0.1300 (4)	0.0887 (15)	
H11	0.7791	−0.0120	0.0669	0.106*	
C12	0.8918 (3)	0.0786 (6)	0.1630 (4)	0.0750 (12)	
H12	0.9365	0.0184	0.1268	0.090*	
C13	0.9071 (4)	0.1987 (7)	0.2598 (5)	0.0973 (18)	
H13	0.9625	0.2337	0.3017	0.117*	
C14	0.8167 (6)	0.2588 (5)	0.2818 (5)	0.123 (3)	
H14	0.8017	0.3416	0.3397	0.148*	
N1	0.61150 (15)	−0.1138 (3)	0.5390 (2)	0.0407 (5)	
N2	0.53352 (17)	−0.1716 (4)	0.6965 (2)	0.0518 (7)	
Fe1	0.83161 (2)	−0.00206 (5)	0.31303 (3)	0.03545 (14)	

### Atomic displacement parameters (Å^2^)

**Table:**

	*U*^11^	*U*^22^	*U*^33^	*U*^12^	*U*^13^	*U*^23^
C1	0.0351 (14)	0.0369 (15)	0.0384 (14)	−0.0031 (12)	0.0057 (11)	0.0063 (11)
C2	0.0457 (17)	0.0350 (15)	0.0472 (16)	−0.0136 (13)	0.0058 (13)	−0.0007 (12)
C3	0.0472 (17)	0.0345 (15)	0.0586 (18)	0.0027 (13)	0.0106 (14)	0.0007 (13)
C4	0.0345 (15)	0.0484 (17)	0.0556 (18)	0.0019 (13)	0.0013 (13)	0.0107 (14)
C5	0.0464 (17)	0.0405 (15)	0.0359 (14)	−0.0038 (13)	0.0016 (12)	0.0027 (11)
C6	0.0422 (15)	0.0494 (18)	0.0523 (17)	0.0031 (14)	0.0142 (13)	0.0135 (14)
C7	0.059 (2)	0.0536 (19)	0.0461 (17)	−0.0088 (15)	0.0189 (15)	−0.0071 (14)
C8	0.0466 (17)	0.063 (2)	0.0465 (17)	−0.0092 (15)	0.0106 (14)	0.0064 (15)
C9	0.0448 (17)	0.0511 (19)	0.0506 (18)	0.0072 (14)	0.0129 (14)	−0.0042 (14)
C10	0.090 (3)	0.097 (4)	0.135 (5)	0.044 (3)	0.049 (3)	0.084 (4)
C11	0.107 (4)	0.097 (3)	0.058 (2)	−0.029 (3)	−0.009 (2)	0.035 (2)
C12	0.088 (3)	0.074 (3)	0.072 (3)	0.008 (2)	0.042 (2)	0.024 (2)
C13	0.107 (4)	0.097 (4)	0.082 (3)	−0.069 (3)	−0.015 (3)	0.041 (3)
C14	0.286 (9)	0.0223 (19)	0.081 (3)	0.009 (3)	0.104 (5)	0.0110 (18)
N1	0.0374 (13)	0.0450 (14)	0.0411 (13)	−0.0004 (11)	0.0108 (10)	0.0031 (11)
N2	0.0479 (15)	0.0667 (18)	0.0434 (14)	0.0045 (13)	0.0161 (12)	0.0017 (13)
Fe1	0.0377 (2)	0.0310 (2)	0.0387 (2)	−0.00352 (17)	0.00896 (15)	0.00289 (17)

### Geometric parameters (Å, °)

**Table:**

C1—C2	1.423 (4)	C8—N2	1.365 (4)
C1—C5	1.424 (4)	C8—H8	0.9300
C1—C6	1.492 (4)	C9—N2	1.311 (4)
C1—Fe1	2.024 (3)	C9—N1	1.343 (4)
C2—C3	1.410 (4)	C9—H9	0.9300
C2—Fe1	2.026 (3)	C10—C11	1.303 (7)
C2—H2	0.9300	C10—C14	1.375 (8)
C3—C4	1.408 (4)	C10—Fe1	1.998 (4)
C3—Fe1	2.040 (3)	C10—H10	0.9300
C3—H3	0.9300	C11—C12	1.305 (6)
C4—C5	1.410 (4)	C11—Fe1	2.026 (4)
C4—Fe1	2.040 (3)	C11—H11	0.9300
C4—H4	0.9300	C12—C13	1.382 (6)
C5—Fe1	2.037 (3)	C12—Fe1	2.035 (3)
C5—H5	0.9300	C12—H12	0.9300
C6—N1	1.463 (3)	C13—C14	1.467 (8)
C6—H6A	0.9700	C13—Fe1	2.013 (3)
C6—H6B	0.9700	C13—H13	0.9300
C7—C8	1.361 (4)	C14—Fe1	2.008 (4)
C7—N1	1.362 (4)	C14—H14	0.9300
C7—H7	0.9300		
			
C2—C1—C5	106.9 (2)	Fe1—C12—H12	126.3
C2—C1—C6	125.5 (3)	C12—C13—C14	104.7 (4)
C5—C1—C6	127.5 (3)	C12—C13—Fe1	70.9 (2)
C2—C1—Fe1	69.49 (15)	C14—C13—Fe1	68.4 (2)
C5—C1—Fe1	69.97 (15)	C12—C13—H13	127.7
C6—C1—Fe1	123.35 (19)	C14—C13—H13	127.7
C3—C2—C1	108.4 (2)	Fe1—C13—H13	124.7
C3—C2—Fe1	70.25 (16)	C10—C14—C13	104.1 (4)
C1—C2—Fe1	69.36 (15)	C10—C14—Fe1	69.5 (2)
C3—C2—H2	125.8	C13—C14—Fe1	68.8 (2)
C1—C2—H2	125.8	C10—C14—H14	128.0
Fe1—C2—H2	126.2	C13—C14—H14	128.0
C4—C3—C2	108.1 (3)	Fe1—C14—H14	125.4
C4—C3—Fe1	69.80 (17)	C9—N1—C7	106.4 (2)
C2—C3—Fe1	69.15 (16)	C9—N1—C6	127.4 (3)
C4—C3—H3	126.0	C7—N1—C6	126.2 (2)
C2—C3—H3	126.0	C9—N2—C8	104.2 (2)
Fe1—C3—H3	126.7	C10—Fe1—C14	40.1 (2)
C3—C4—C5	108.3 (3)	C10—Fe1—C13	68.0 (2)
C3—C4—Fe1	69.82 (17)	C14—Fe1—C13	42.8 (2)
C5—C4—Fe1	69.66 (16)	C10—Fe1—C1	107.71 (16)
C3—C4—H4	125.9	C14—Fe1—C1	113.20 (19)
C5—C4—H4	125.9	C13—Fe1—C1	148.6 (2)
Fe1—C4—H4	126.2	C10—Fe1—C2	112.3 (2)
C4—C5—C1	108.3 (3)	C14—Fe1—C2	143.2 (3)
C4—C5—Fe1	69.87 (17)	C13—Fe1—C2	170.3 (2)
C1—C5—Fe1	68.97 (15)	C1—Fe1—C2	41.15 (11)
C4—C5—H5	125.9	C10—Fe1—C11	37.8 (2)
C1—C5—H5	125.9	C14—Fe1—C11	66.1 (2)
Fe1—C5—H5	126.9	C13—Fe1—C11	65.89 (18)
N1—C6—C1	113.1 (2)	C1—Fe1—C11	130.50 (18)
N1—C6—H6A	109.0	C2—Fe1—C11	107.92 (16)
C1—C6—H6A	109.0	C10—Fe1—C12	64.48 (18)
N1—C6—H6B	109.0	C14—Fe1—C12	67.83 (18)
C1—C6—H6B	109.0	C13—Fe1—C12	39.91 (19)
H6A—C6—H6B	107.8	C1—Fe1—C12	167.51 (17)
C8—C7—N1	105.8 (3)	C2—Fe1—C12	130.71 (17)
C8—C7—H7	127.1	C11—Fe1—C12	37.50 (18)
N1—C7—H7	127.1	C10—Fe1—C5	134.2 (2)
C7—C8—N2	110.7 (3)	C14—Fe1—C5	110.93 (16)
C7—C8—H8	124.7	C13—Fe1—C5	118.80 (16)
N2—C8—H8	124.7	C1—Fe1—C5	41.06 (11)
N2—C9—N1	113.0 (3)	C2—Fe1—C5	68.54 (12)
N2—C9—H9	123.5	C11—Fe1—C5	170.4 (2)
N1—C9—H9	123.5	C12—Fe1—C5	151.27 (17)
C11—C10—C14	110.5 (5)	C10—Fe1—C4	174.5 (2)
C11—C10—Fe1	72.3 (3)	C14—Fe1—C4	136.4 (3)
C14—C10—Fe1	70.3 (3)	C13—Fe1—C4	112.53 (16)
C11—C10—H10	124.8	C1—Fe1—C4	68.85 (11)
C14—C10—H10	124.8	C2—Fe1—C4	68.27 (12)
Fe1—C10—H10	124.2	C11—Fe1—C4	147.7 (2)
C10—C11—C12	111.1 (5)	C12—Fe1—C4	119.68 (16)
C10—C11—Fe1	69.9 (3)	C5—Fe1—C4	40.47 (12)
C12—C11—Fe1	71.6 (2)	C10—Fe1—C3	143.3 (2)
C10—C11—H11	124.4	C14—Fe1—C3	175.9 (3)
C12—C11—H11	124.4	C13—Fe1—C3	133.8 (2)
Fe1—C11—H11	125.6	C1—Fe1—C3	68.89 (12)
C11—C12—C13	109.7 (5)	C2—Fe1—C3	40.59 (12)
C11—C12—Fe1	70.9 (2)	C11—Fe1—C3	115.5 (2)
C13—C12—Fe1	69.2 (2)	C12—Fe1—C3	110.95 (16)
C11—C12—H12	125.2	C5—Fe1—C3	68.13 (12)
C13—C12—H12	125.2	C4—Fe1—C3	40.37 (12)
			
C5—C1—C2—C3	−0.7 (3)	C5—C1—Fe1—C11	−173.4 (3)
C6—C1—C2—C3	176.6 (3)	C6—C1—Fe1—C11	−51.0 (4)
Fe1—C1—C2—C3	59.6 (2)	C2—C1—Fe1—C12	54.4 (7)
C5—C1—C2—Fe1	−60.26 (19)	C5—C1—Fe1—C12	172.2 (7)
C6—C1—C2—Fe1	117.0 (3)	C6—C1—Fe1—C12	−65.4 (8)
C1—C2—C3—C4	0.1 (3)	C2—C1—Fe1—C5	−117.8 (2)
Fe1—C2—C3—C4	59.1 (2)	C6—C1—Fe1—C5	122.4 (3)
C1—C2—C3—Fe1	−59.04 (19)	C2—C1—Fe1—C4	−80.75 (18)
C2—C3—C4—C5	0.5 (3)	C5—C1—Fe1—C4	37.09 (17)
Fe1—C3—C4—C5	59.3 (2)	C6—C1—Fe1—C4	159.5 (3)
C2—C3—C4—Fe1	−58.7 (2)	C2—C1—Fe1—C3	−37.33 (17)
C3—C4—C5—C1	−0.9 (3)	C5—C1—Fe1—C3	80.52 (19)
Fe1—C4—C5—C1	58.42 (19)	C6—C1—Fe1—C3	−157.1 (3)
C3—C4—C5—Fe1	−59.4 (2)	C3—C2—Fe1—C10	148.6 (3)
C2—C1—C5—C4	1.0 (3)	C1—C2—Fe1—C10	−91.8 (3)
C6—C1—C5—C4	−176.2 (3)	C3—C2—Fe1—C14	−177.6 (3)
Fe1—C1—C5—C4	−59.0 (2)	C1—C2—Fe1—C14	−58.0 (3)
C2—C1—C5—Fe1	59.95 (18)	C3—C2—Fe1—C1	−119.6 (2)
C6—C1—C5—Fe1	−117.2 (3)	C3—C2—Fe1—C11	108.5 (3)
C2—C1—C6—N1	89.2 (3)	C1—C2—Fe1—C11	−131.9 (2)
C5—C1—C6—N1	−94.1 (3)	C3—C2—Fe1—C12	73.8 (3)
Fe1—C1—C6—N1	176.54 (19)	C1—C2—Fe1—C12	−166.6 (2)
N1—C7—C8—N2	0.1 (4)	C3—C2—Fe1—C5	−81.00 (19)
C14—C10—C11—C12	0.3 (5)	C1—C2—Fe1—C5	38.61 (16)
Fe1—C10—C11—C12	−59.9 (3)	C3—C2—Fe1—C4	−37.32 (18)
C14—C10—C11—Fe1	60.2 (3)	C1—C2—Fe1—C4	82.29 (18)
C10—C11—C12—C13	0.5 (5)	C1—C2—Fe1—C3	119.6 (2)
Fe1—C11—C12—C13	−58.5 (3)	C12—C11—Fe1—C10	121.7 (5)
C10—C11—C12—Fe1	58.9 (3)	C10—C11—Fe1—C14	−37.5 (4)
C11—C12—C13—C14	−1.0 (5)	C12—C11—Fe1—C14	84.2 (4)
Fe1—C12—C13—C14	−60.5 (2)	C10—C11—Fe1—C13	−84.6 (4)
C11—C12—C13—Fe1	59.5 (3)	C12—C11—Fe1—C13	37.1 (3)
C11—C10—C14—C13	−0.9 (5)	C10—C11—Fe1—C1	63.3 (4)
Fe1—C10—C14—C13	60.5 (3)	C12—C11—Fe1—C1	−174.9 (3)
C11—C10—C14—Fe1	−61.4 (3)	C10—C11—Fe1—C2	103.5 (4)
C12—C13—C14—C10	1.1 (4)	C12—C11—Fe1—C2	−134.8 (3)
Fe1—C13—C14—C10	−61.0 (3)	C10—C11—Fe1—C12	−121.7 (5)
C12—C13—C14—Fe1	62.1 (3)	C10—C11—Fe1—C4	−179.0 (3)
N2—C9—N1—C7	0.6 (4)	C12—C11—Fe1—C4	−57.3 (5)
N2—C9—N1—C6	177.8 (3)	C10—C11—Fe1—C3	146.6 (3)
C8—C7—N1—C9	−0.4 (3)	C12—C11—Fe1—C3	−91.7 (3)
C8—C7—N1—C6	−177.6 (3)	C11—C12—Fe1—C10	−35.3 (4)
C1—C6—N1—C9	127.7 (3)	C13—C12—Fe1—C10	85.6 (4)
C1—C6—N1—C7	−55.7 (4)	C11—C12—Fe1—C14	−79.2 (4)
N1—C9—N2—C8	−0.5 (4)	C13—C12—Fe1—C14	41.6 (3)
C7—C8—N2—C9	0.3 (4)	C11—C12—Fe1—C13	−120.8 (5)
C11—C10—Fe1—C14	120.3 (5)	C11—C12—Fe1—C1	18.1 (9)
C11—C10—Fe1—C13	78.7 (3)	C13—C12—Fe1—C1	138.9 (7)
C14—C10—Fe1—C13	−41.6 (3)	C11—C12—Fe1—C2	63.0 (4)
C11—C10—Fe1—C1	−134.5 (3)	C13—C12—Fe1—C2	−176.2 (3)
C14—C10—Fe1—C1	105.2 (3)	C13—C12—Fe1—C11	120.8 (5)
C11—C10—Fe1—C2	−90.8 (3)	C11—C12—Fe1—C5	−172.6 (3)
C14—C10—Fe1—C2	148.9 (3)	C13—C12—Fe1—C5	−51.8 (5)
C14—C10—Fe1—C11	−120.3 (5)	C11—C12—Fe1—C4	148.8 (3)
C11—C10—Fe1—C12	35.0 (3)	C13—C12—Fe1—C4	−90.3 (3)
C14—C10—Fe1—C12	−85.3 (3)	C11—C12—Fe1—C3	105.0 (3)
C11—C10—Fe1—C5	−172.0 (3)	C13—C12—Fe1—C3	−134.2 (3)
C14—C10—Fe1—C5	67.7 (4)	C4—C5—Fe1—C10	−178.0 (3)
C11—C10—Fe1—C3	−56.3 (4)	C1—C5—Fe1—C10	62.0 (3)
C14—C10—Fe1—C3	−176.6 (3)	C4—C5—Fe1—C14	−138.3 (3)
C13—C14—Fe1—C10	−115.0 (4)	C1—C5—Fe1—C14	101.7 (3)
C10—C14—Fe1—C13	115.0 (4)	C4—C5—Fe1—C13	−91.7 (3)
C10—C14—Fe1—C1	−90.3 (3)	C1—C5—Fe1—C13	148.4 (3)
C13—C14—Fe1—C1	154.7 (3)	C4—C5—Fe1—C1	119.9 (2)
C10—C14—Fe1—C2	−52.9 (4)	C4—C5—Fe1—C2	81.24 (19)
C13—C14—Fe1—C2	−168.0 (2)	C1—C5—Fe1—C2	−38.70 (17)
C10—C14—Fe1—C11	35.4 (3)	C4—C5—Fe1—C12	−56.6 (4)
C13—C14—Fe1—C11	−79.7 (3)	C1—C5—Fe1—C12	−176.5 (3)
C10—C14—Fe1—C12	76.2 (3)	C1—C5—Fe1—C4	−119.9 (2)
C13—C14—Fe1—C12	−38.8 (3)	C4—C5—Fe1—C3	37.41 (17)
C10—C14—Fe1—C5	−134.7 (3)	C1—C5—Fe1—C3	−82.53 (18)
C13—C14—Fe1—C5	110.3 (3)	C3—C4—Fe1—C14	−176.2 (3)
C10—C14—Fe1—C4	−173.5 (3)	C5—C4—Fe1—C14	64.3 (3)
C13—C14—Fe1—C4	71.5 (3)	C3—C4—Fe1—C13	−132.0 (3)
C12—C13—Fe1—C10	−76.1 (3)	C5—C4—Fe1—C13	108.5 (3)
C14—C13—Fe1—C10	39.1 (3)	C3—C4—Fe1—C1	81.89 (19)
C12—C13—Fe1—C14	−115.2 (4)	C5—C4—Fe1—C1	−37.61 (17)
C12—C13—Fe1—C1	−164.2 (3)	C3—C4—Fe1—C2	37.52 (17)
C14—C13—Fe1—C1	−49.0 (4)	C5—C4—Fe1—C2	−81.98 (18)
C12—C13—Fe1—C11	−34.9 (3)	C3—C4—Fe1—C11	−51.9 (4)
C14—C13—Fe1—C11	80.2 (3)	C5—C4—Fe1—C11	−171.4 (3)
C14—C13—Fe1—C12	115.2 (4)	C3—C4—Fe1—C12	−88.0 (2)
C12—C13—Fe1—C5	154.5 (3)	C5—C4—Fe1—C12	152.5 (2)
C14—C13—Fe1—C5	−90.4 (3)	C3—C4—Fe1—C5	119.5 (3)
C12—C13—Fe1—C4	109.9 (3)	C5—C4—Fe1—C3	−119.5 (3)
C14—C13—Fe1—C4	−135.0 (3)	C4—C3—Fe1—C10	−173.4 (3)
C12—C13—Fe1—C3	68.0 (3)	C2—C3—Fe1—C10	−53.8 (3)
C14—C13—Fe1—C3	−176.8 (3)	C4—C3—Fe1—C13	72.0 (3)
C2—C1—Fe1—C10	103.8 (3)	C2—C3—Fe1—C13	−168.4 (2)
C5—C1—Fe1—C10	−138.3 (3)	C4—C3—Fe1—C1	−81.78 (19)
C6—C1—Fe1—C10	−15.9 (3)	C2—C3—Fe1—C1	37.83 (17)
C2—C1—Fe1—C14	146.4 (3)	C4—C3—Fe1—C2	−119.6 (3)
C5—C1—Fe1—C14	−95.7 (3)	C4—C3—Fe1—C11	152.3 (2)
C6—C1—Fe1—C14	26.7 (4)	C2—C3—Fe1—C11	−88.1 (2)
C2—C1—Fe1—C13	−179.6 (3)	C4—C3—Fe1—C12	111.6 (2)
C5—C1—Fe1—C13	−61.8 (4)	C2—C3—Fe1—C12	−128.8 (2)
C6—C1—Fe1—C13	60.6 (4)	C4—C3—Fe1—C5	−37.50 (18)
C5—C1—Fe1—C2	117.8 (2)	C2—C3—Fe1—C5	82.10 (18)
C6—C1—Fe1—C2	−119.7 (3)	C2—C3—Fe1—C4	119.6 (3)
C2—C1—Fe1—C11	68.7 (3)		
